# Publisher Correction: Superplasticity in a lean Fe-Mn-Al steel

**DOI:** 10.1038/s41467-017-02132-9

**Published:** 2018-01-10

**Authors:** Jeongho Han, Seok-Hyeon Kang, Seung-Joon Lee, Megumi Kawasaki, Han-Joo Lee, Dirk Ponge, Dierk Raabe, Young-Kook Lee

**Affiliations:** 1 Department of Materials Science and Engineering, Yonsei University, Seoul, 03722 Republic of Korea; 2 Division of Materials Science and Engineering, Hanyang University, Seoul, 04763 Republic of Korea; 3 Max-Planck-Institut für Eisenforschung, Max-Planck-Straβe 1, Düsseldorf, 40237 Germany; 4 Present Address: Department of Materials Science and Engineering, Chungnam National University, Daejeon, 34134 Republic of Korea; 5 Present Address: Joining and Welding Research Institute, Osaka University, Osaka, 567-0047 Japan

Correction to: *Nature Communications* 10.1038/s41467-017-00814-y, published online 29 Sep 2017

The original PDF version of this Article omitted to state that “Jeongho Han and Seok-Hyeon Kang contributed equally to this work” in the affiliations section. This has now been corrected in the PDF version of the Article. The HTML version was correct from the time of publication.

