# Supplementary material for: Superplasticity in a lean Fe-Mn-Al steel
Source: Nat Commun. 2017 Sep 29;8:751. doi: 10.1038/s41467-017-00814-y (PMC5622104; doi:10.1038/s41467-017-00814-y)
Supplement: Supplementary file 1 — Supplementary Information [file 41467_2017_814_MOESM1_ESM.pdf]

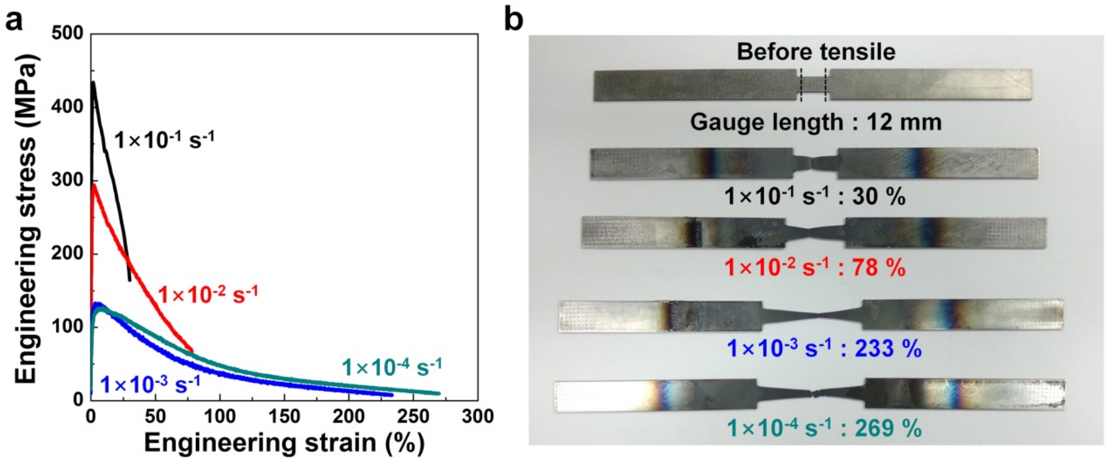

Supplementary Figure 1. Tensile properties of Fe-7Mn-0.05C (wt.%) steel strained at 645 °C with various initial strain rates. (**a**) Engineering stress-strain curves. (**b**) Images of fractured tensile specimens.

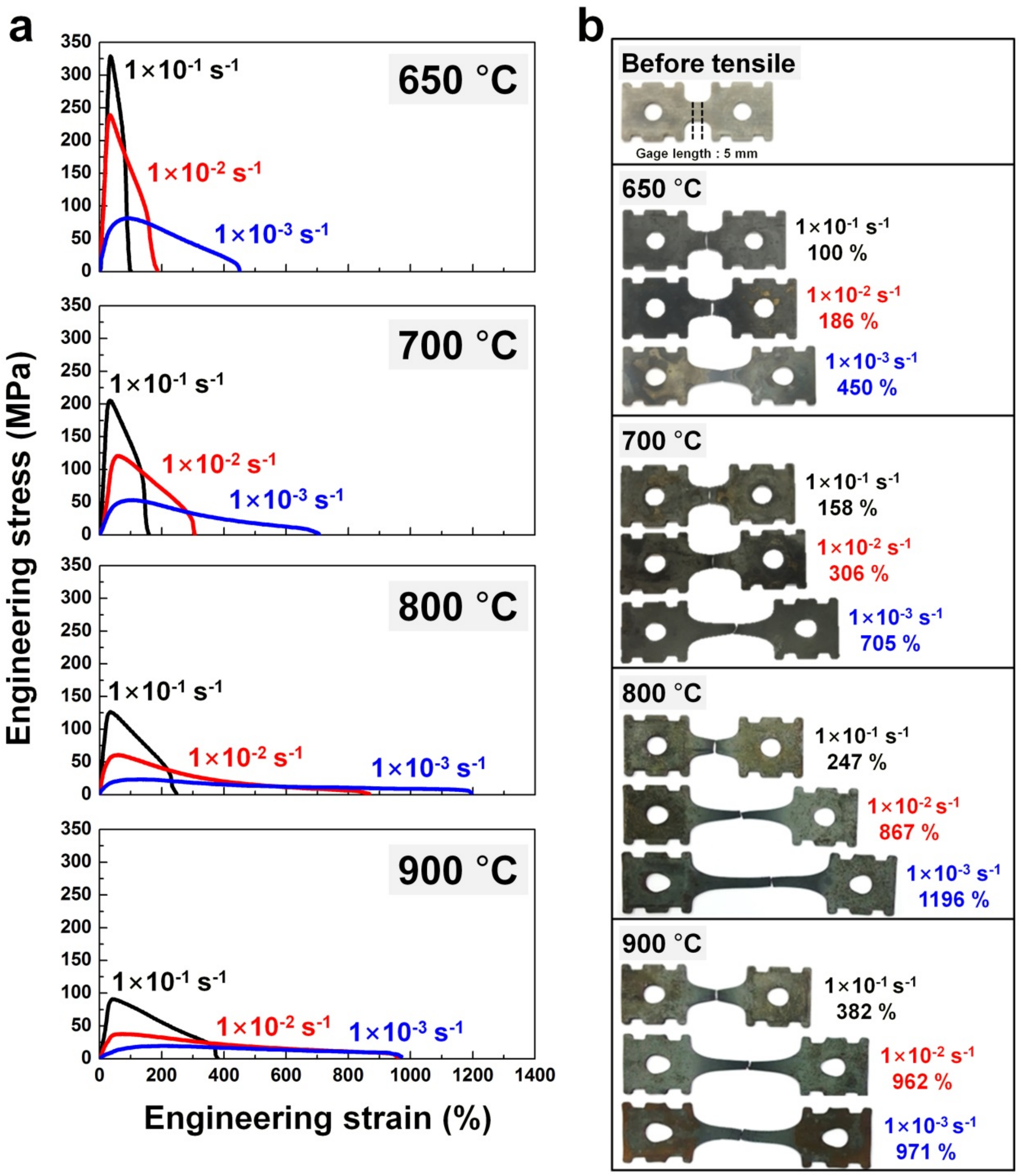

Supplementary Figure 2. Tensile properties of Fe-6.6Mn-2.3Al (wt.%) steel strained at temperatures from 650 °C to 900 °C with various initial strain rates. (**a**) Engineering stress-strain curves. (**b**) Images of fractured tensile specimens.

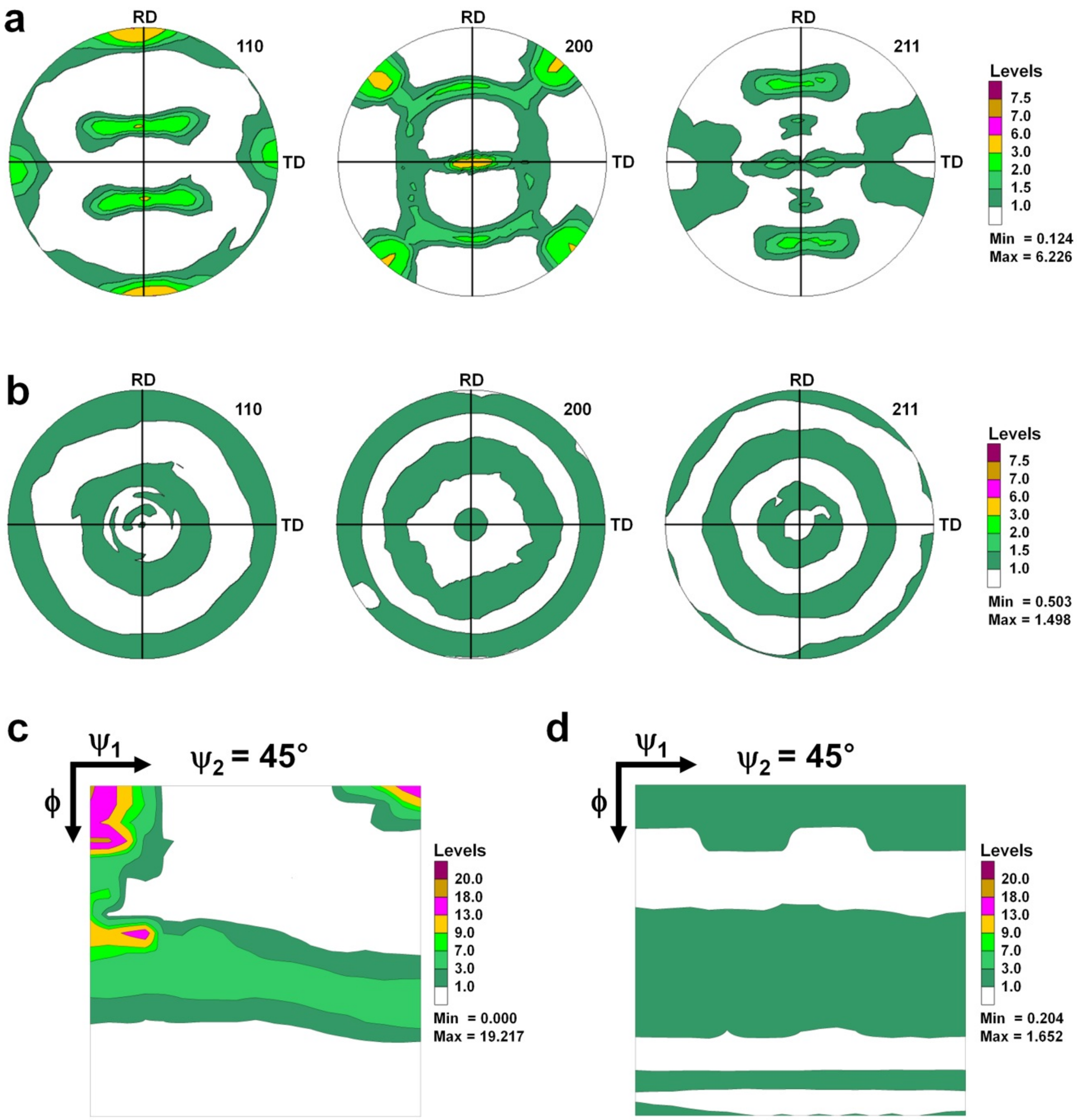

Supplementary Figure 3. Change in overall texture of Fe-6.6Mn-2.3Al (wt. %) steel before and after the tensile test at 850 °C at an initial strain rate of 1.0 × 10^{-3} s^{-1}. (**a**) Pole figure before the tensile test. (**b**) Pole figure after the tensile test. (**c**) Orientation distribution function before the tensile test. (**d**) Orientation distribution function after the tensile test.

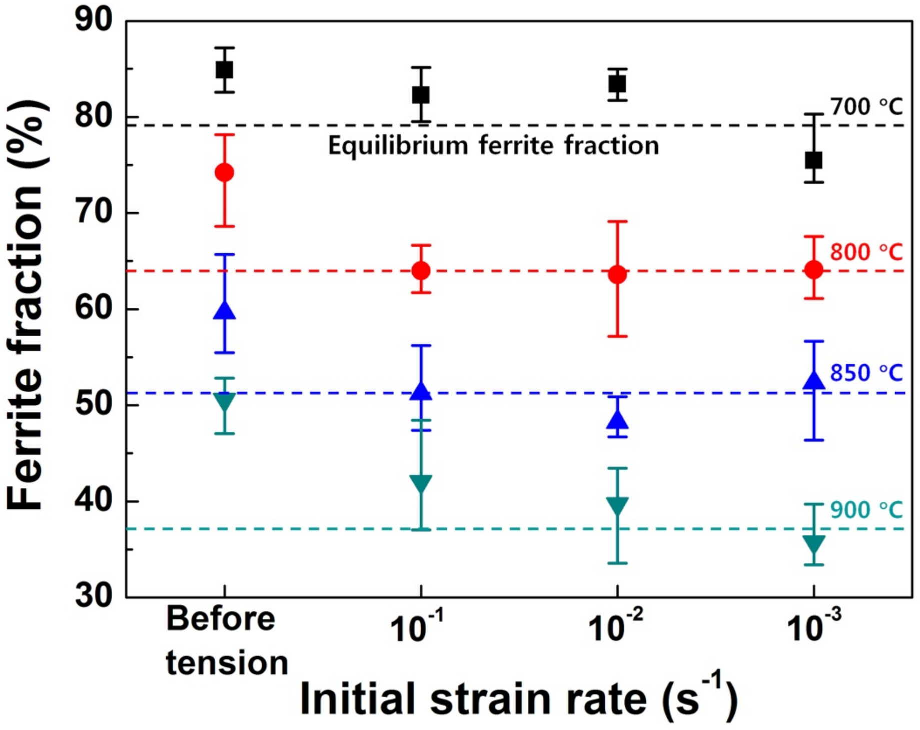

Supplementary Figure 4. Variation of ferrite fraction with initial strain rate and tensile deformation temperature in Fe-6.6Mn-2.3Al (wt. %) steel.

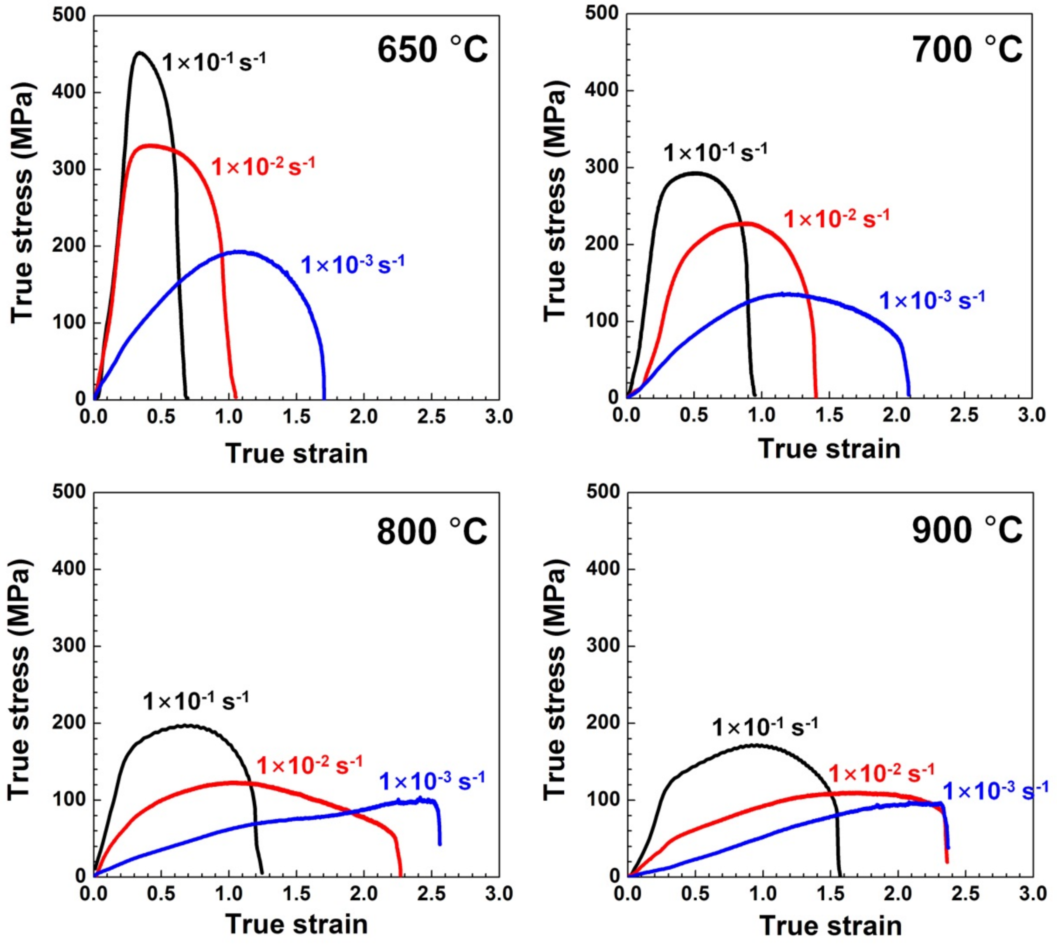

Supplementary Figure 5. True stress-strain curves of Fe-6.6Mn-2.3Al (wt. %) steel strained at temperatures from 650 °C and 900 °C with various initial strain rate from 1.0×10^{-3} s^{-1} to 1.0×10^{-1} s^{-1}.

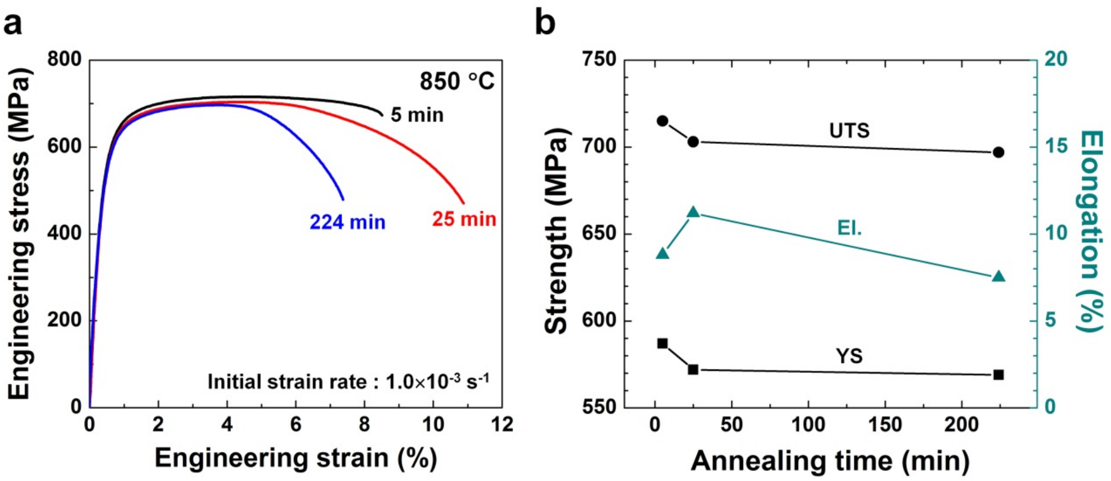

Supplementary Figure 6. Variation of room-temperature tensile properties with annealing time at 850 °C in Fe-6.6Mn-2.3Al (wt. %) steel. (**a**) Engineering stress-strain curves measured at the initial strain rate of 1.0×10^{-3} s^{-1}. (**b**) Yield strength, ultimate tensile strength, and elongation.

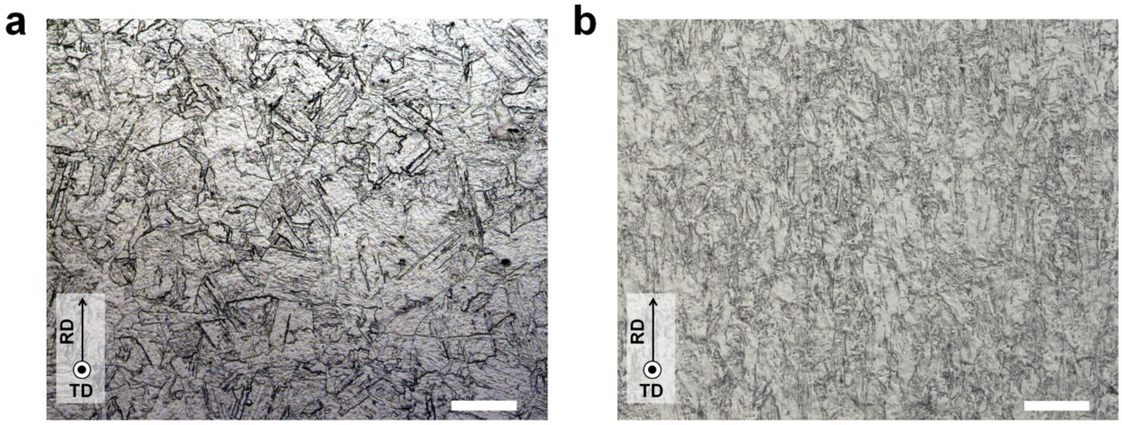

Supplementary Figure 7. Optical microstructures of Fe-6.6Mn-2.3Al (wt. %) steel. (**a**) Hot-rolled specimen. Scale bar, 100 μm. (**b**) Cold-rolled specimen. Scale bar, 100 μm.

Supplementary Table 1. Chemical composition, thermomechanical pre-treatment and elongation and deformation conditions of superplastic materials

Alloy	Chemical composition (wt.%)	Thermomechanical pre-treatment	Elongation (%)	Temperature (°C)	Strain rate (s^{-1})	Ref.
IC-218	8.5Al-7.8Cr-0.8Zr-0.02B	Two batch annealing	375	650	1.0×10^{-3}	7
			302	950	8.3×10^{-4}	
			352	1000		
			437	1050		
			469	1100		
			638	1100		
Ti-64	6Al-4V	Multi stage forging	700	775	1.0×10^{-3}	12
			850		1.0×10^{-4}	
			380	875	1.0×10^{-2}	
			390		1.0×10^{-3}	
			720			
			420		1.0×10^{-4}	
			720			
Duplex stainless steel	26Cr-6.5Ni-0.4Ti	Hot + cold rolling	600	960	1.6×10^{-3}	13
			820		8.0×10^{-4}	
			1050		3.2×10^{-4}	
			890		1.6×10^{-4}	
	25Cr-7Ni-3Mo-0.14N		574	850	2.0×10^{-3}	14
			1100	900		
			2500	950		
			900	1000	1.7×10^{-1}	16
			1700		1.7×10^{-2}	
			700		1.7×10^{-3}	
	22Cr-5Ni-3Mo-0.18N		1510	980	3.0×10^{-4}	17
	18.5Cr-4Ni-1.7Si-3.2Mn-1.2Cu		750	950	2.0×10^{-3}	15
			620		1.0×10^{-3}	
Ultra high carbon steel	1.3C-0.7Mn-0.1Si	Hot + warm rolling	700	630	1.3×10^{-4}	18
			480	650	1.7×10^{-4}	
			600	650	1.3×10^{-4}	
	1.6C-0.7Mn-0.3Si		489	620	6.7×10^{-5}	
			760	630	1.3×10^{-4}	
			473	650	6.7×10^{-5}	
	1.9C-0.8Mn-0.3Si		380	650	1.7×10^{-4}	
	0.9C-1.2Mn-0.5Cr-0.5W-0.2V	Warm rolling + thermal cycling	1200	650	1.6×10^{-4}	19
	1.3C-3.0Si-1.5Cr-0.5Mn	Hot forging + two step warm rolling	515	850	1.7×10^{-2}	20
			942		1.0×10^{-2}	
			1300		1.7×10^{-4}	
	1.3C-2.3Mn-1.8Si-1.1Cr-1Al	Hot rolling + annealing + warm rolling	525	700	2.0×10^{-3}	21
			570	730		
			720	780		
Medium Mn steel	6.6Mn-2.3Al	Hot + cold rolling	450	650	1.0×10^{-3}	-
			306	700	1.0×10^{-2}	
			705		1.0×10^{-3}	
			867	800	1.0×10^{-2}	
			1196		1.0×10^{-3}	
			1113	850	1.0×10^{-2}	
			1314		1.0×10^{-3}	
			382	900	1.0×10^{-1}	
			962		1.0×10^{-2}	
			971		1.0×10^{-3}	
